# In vitro production of functional immune cells derived from human hematopoietic stem cells

**DOI:** 10.17179/excli2015-506

**Published:** 2015-09-09

**Authors:** Witchuda Payuhakrit, Tasanee Panichakul, Natthawut Charoenphon, Panus Chalermsaenyakorn, Adithep Jaovisidha, Chokdee Wongborisuth, Rachanee Udomsangpetch

**Affiliations:** 1 Department of Pathobiology, Faculty of Science, Mahidol University, Thailand; 2 Faculty of Science and Technology, Suan Dusit Rajabhat University, Thailand; 3 Department of Pathology, Faculty of Medicine Ramathibodi Hospital, Thailand; 4 Department of Obstetrics and Gynaecology, Faculty of Medicine, Ramathibodi Hospital, Mahidol University, Thailand; 5 Research Center, Faculty of Medicine, Ramathibodi Hospital, Mahidol University, Thailand; 6 Center for Research and Innovation, Faculty of Medical Technology, Mahidol University, Thailand; 7 Center for Emerging and Neglected Infectious Diseases, Mahidol University, Thailand

**Keywords:** hematopoietic stem cells, stem cells, lymphocyte maturation, myelopoietic cells

## Abstract

Hematopoietic stem cells (HSC) from cord blood are potentially high sources for transplantation due to their low immunogenicity and the presence of the multipotent cells. These cells are capable of differentiating to produce various lineages of blood cells under specific conditions. We have enriched highly purified CD34^+^ cells from cord blood, determined in vitro growth of the cells in culture systems in the absence (condition A) or presence of GM-CSF and G-CSF (condition B), and determined the profile of immune cells during the period of cultivation by using flow cytometry. PhytohemagglutininA (PHA) was used as a mitogen to stimulate T lymphocytes derived from hematopoietic stem cells. GM-CSF and G-CSF prolonged the survival of the growing cells and also maintained expansion of cells in blastic stage. By day 12 of cultivation, when cell numbers peaked, various types of immune cells had appeared (CD14^+^ cells, CD40^+^HLA-DR^+^ cells, CD3^+^CD56^+^ cells, CD19^+^ cells, CD3^+^CD4^+ ^cells, CD3^+^CD8^+^cells and CD3-CD56^+^). A significantly higher percentage of monocytes (p = 0.002) were observed under culture with GM-CSF, G-CSF when compared with culture without GM-CSF, G-CSF. In addition, T lymphocytes derived from HSC responded to 50 µg/ml of PHA. This is the first report showing the complete differentiation and proliferation of immune cells derived from CD34^+^ HSC under in vitro culture conditions. Lymphocytes, monocytes, dendritic cells and polymorph nuclear cells derived from HSC in vitro are unique, and thus may benefit various studies such as innate immunity and pathophysiology of immune disorders.

## Introduction

HSC derived from umbilical cord blood have the potential for use in transplantation due to their low immunogenicity. These pluripotent cells are capable of differentiation to produce various lineages of blood cells under specific conditions (Newcomb et al., 2007[21]) and CD34^+^ HSC can be driven to become either lymphoid or myeloid cells *in vitro *(Huang and Terstappen, 1994[12]). Blood cells such as neutrophils and platelets derived from *in vitro* differentiated HSC have been investigated for use in cellular immunotherapy (Timmins et al., 2009[24]; Chen et al., 2009[7]). However, *in vitro* development of lymphoid lineages and the immune functions of these cells have only rarely been explored.

Hematopoietic growth factors i.e. stem cell factor (SCF) (Hassan and Zander, 1996[11]), IL-3 (Bryder and Jacobsen, 2000[4]), and colony-stimulating factor (CSF) (Bociek and Armitage, 1996[3]; Clark and Kamen, 1987[8]), are crucial stimulators that drive the differentiation of HSC to various cell types (Kaushansky, 2006[14]). The multiple benefits for each of these hematopoietic growth factors have been well characterized and *in vitro* analysis has revealed synergistic effects of SCF, IL-1, IL3 and IL-6 on hematopoietic progenitor cells (Leary et al., 1988[17]; Duarte and Frank, 2000[10]).

Generally, *in vitro* analysis of cellular immune responses to antigens employs peripheral blood mononuclear cells (PBMC); *in vitro* vaccine testing also relies on PBMC activation and proliferation (Castle, 1994[6]). However, cells that are exposed to antigens in the human body are not only the circulating immune competent cells but also the newly differentiated and young progenitor cells that are found in bone marrow and may not be in peripheral blood. Evidence supporting the ability of progenitor cells to respond to signals comes from the presence of Toll-like receptors and their co-stimulatory molecules on the multi-potential HSC (Nagai et al., 2006[20]). For the antigen-experienced adult, memory Tcells (CD45RO^+^) comprise around 50 % of T cells in peripheral blood with the proportion increasing with age to approximately 80 % of circulating T cells in centenarians (Cossarizza et al., 1996[9]). Based on these findings naïve T lymphocytes are relatively rare in peripheral blood and how these newly differentiated T lymphocytes respond to antigenic stimulation is not known.

This study characterizes the phenotypes and functions of various immune cells generated from the *in vitro* differentiation of CD34^+^ HSC as well as the phenotypic profiles of cell proliferation Moreover, mitogenic responsiveness of the HSC-derived T lymphocytes from CD34^+^ cells are also compared and discussed.

## Materials and Methods

### Purification of cord blood CD34^+^ cells

Umbilical cord blood was obtained from mothers at normal full-term delivery with informed consent. Cord blood was collected in the sterile blood collection bags containing 30 ml of citrate phosphate dextrose (Kawasumi Laboratories, Thailand, Co., LTD) as an anticoagulant, and processed within 4 h. Mononuclear cells (MNCs) were separated by density gradient centrifugation (1,200 g for 20 min at 20 °C) using LymphoPrep™ (Axis-Shield PoC AS, Oslo, Norway). The mononuclear cell fraction was collected and washed twice with cool PBS containing 2mM EDTA. A CD34 isolation kit utilizing the Mini-MACS magnetic microbead selection (Miltenyi Biotech, Germany) was used to enrich CD34^+^ cells from the MNCs population. The isolated CD34^+^ cells were serially passed through two Mini-MACS columns to increase the purity of CD34^+^ cells and eliminate contaminating mature cells. The purity of isolated CD34^+ ^HSC was determined by flow cytometry and viability of the cells was measured using trypan blue exclusion dye staining.

### In vitro cultivation of HSC

To observe the effect of GM-CSF and G-CSF on the proliferation and differentiation of purified CD34^+^ HSC, cells were cultured at a density of 1x10^6^ cells/ml using 12-well tissue culture plates in two different conditions (A and B). Condition A consisted of Stem line II serum-free hematopoietic stem cell expansion medium (Sigma-Aldrich Corporation, Missouri, USA) supplemented with 50 ng/ml stem cell factor (SCF) (PeproTech, Rocky Hill, NJ, USA) , 10 ng/ml IL-3 (R&D Systems, Inc., MN, USA), 100 μg/ml transferrin (Sigma-Aldrich) and 100 μg/ml Humulin® (Lilly Pharma, Giessen, Germany). Condition B was identical to condition A except for the addition of 10 ng/ml GM-CSF and 10 ng/ml G-CSF (PeproTech, Rocky Hill, NJ, USA). Cells were cultured at 37 °C in a humidified atmosphere with 5 % CO_2_. On days 4, 8, 12, 16, and 20 of cultivation, the phenotypes of the cells were determined by flow cytometry using various monoclonal antibodies. Half of the medium was replaced every 4 days and viability was checked every four days with trypan blue exclusion dye staining. 

### Morphological examination

Cells from cultures were stained with Giemsa dye for determining morphology at 12, 16, 20 and 24 days after cultivation. 5-10 x 10^4^ cells in 200 μl of medium were spun onto a slide by cytospin (Cytospin3, Thermo Shandon, UK.), at 400 g for 10 min, and then fixed with 95 % ethanol for 10 min. Thereafter, cells were stained for 10 min with Giemsa diluted with PBS (ratio 1:4 by volume), and washed with tap water. Stained slides were dried at room temperature and the cell morphology was observed under a light microscope. Approximately 300 cells per slide were scored.

### Flow cytometric analysis

The immune phenotypes of cells were determined by flow cytometry to monitor the level of HSC differentiation and the presence of various cell types. Mononuclear cells (1 x 10^5^ cells) from each culture condition were stained with the fluorescent dye-labeled monoclonal antibodies and incubated for 30 minutes at 4 °C in the dark. The cells were then washed twice with PBS by centrifugation at 800 g, 5 min at 4 °C and fixed with 1 % paraformaldehyde in PBS. The stained cells were analyzed by FACScan flow cytometry (Beckman Coulter, USA) to determine the expression pattern of lineage-associated antigens that indicate the various committed cellular subsets using a commercially available kit with fluorochrome-labelled monoclonal antibodies [(Biolegend): CD34^+^HLA-DR^-^, CD3^+^CD4^+^, CD3^+^CD8^+^, CD19^+^, CD14^+^, CD56^+^CD3^-^, CD56^+^CD3^+^, CD40^+^HLA-DR^+^].

### Lymphocyte proliferation assay

In order to confirm the responsiveness of the lymphocytes derived from the cultivated hematopoietic stem cells, the CD34^+^ HSC were expanded under conditions A or B for 10 days in order to obtain a sufficient quantity of cells for the study. Mononuclear cells (2 x 10^5^ cells) were suspended in 100 µl of the culture medium in duplicate wells of 96-well tissue culture plates (Corning Incorporated Costar^®^, NY, USA). The cells were co-cultured with 100 µl PHA at a final concentration of 2, 10, 50 µg/ml, or without PHA. PBMC from 7 healthy donors were isolated according to the protocol previously published by Le and colleagues (2006) and also assayed to compare the level of PHA stimulation. Plates were incubated in a 5 % CO_2 _humidified atmosphere at 37 °C. Cells were harvested on days 2, 4 and 6 of cultivation, washed with PBS, counted viable cells by trypan blue exclusion dye staining. The results were expressed as the mean of duplicate wells and compared with that of control cells cultured without PHA.

### Statistical analysis

Data were obtained from 7 different occasions and analyzed using the SPSS program (version 11.0, Chicago, USA) and results were reported as the means ± SD. Comparisons were analyzed by the nonparametric Mann-Whitney U test to evaluate statistical significance; *p *< 0.05 was considered significant.

## Results

### Isolation and cultivation of CD34^+ ^HSC 

**Figure 1:**
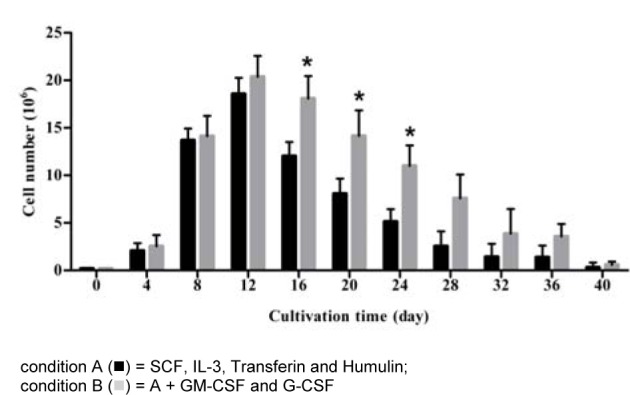
Total nucleated cells expanded from highly purified CD34^+ ^cells under two different conditioned media CD34^+^ cells were cultured in two different media with and without cytokine supplement. Viable cells were counted by trypan blue exclusion method every 4 days after start of cultivation. The data obtained from 7 cord bloods, columns are mean values and bars represent SD. * *p *< 0.05

The amounts of CD34^+^ cells isolated from cord blood ranged from 5 x 10^5^ to 1.1 x 10^6^ cells. Purity of CD34^+^ cells isolated from cord blood mononuclear cells was between 95 and 97 %. In the CD34^+^-enriched MNCs, no differentiated cells were observed upon staining with a wide range of markers for mature immune cells, i.e. CD3, CD4, CD8, CD14, CD19, CD40, and CD56. These cells were cultured with HSC complete medium alone (A condition) or with the addition of GM-CSF and G-CSF (B condition) for 40 days. Growth of the cells is shown in Figure 1(Fig. 1). Total nucleated cells (TNC) had increased 10-fold on day 4 and 70-fold on day 8, and 102-fold on day 12 after cultivation in condition A and 93-fold on day 12 (day of peak) in B conditions. The number of cells present on days 16, 20 and 24 under B condition was significantly higher than that under A condition (*p* = 0.02). These results demonstrated that the total nucleated cells were significantly increased in the presence of GM-CSF and G-CSF (B condition).

### Morphological examination by microscopy

In this study, three cellular lineages were assessed: granulocytic, monocytic, and lymphocytic lineages as shown in Table 1(Tab. 1). There was no significant difference observed of thegranulocyte nor lymphocyte in those two culture conditions. In B condition (day 12), blast cells were significantly higher than that A condition (*p* = 0.02). The numbers of cells in blastic stage were dramatically reduced on day 16 in both culture conditions and were reduced further to undetectable levels by day 20. The numbers of monocytes on days 16, 20 and 24 under B condition were significantly higher than those under A condition (*p* = 0.02). These results indicate that B condition promoted the differentiation of monocytes.

### Cell viability

**Figure 2:**
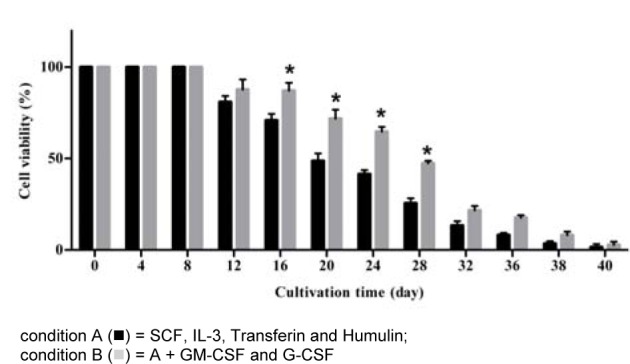
Viability of the growing cells (percentage) derived from hematopoietic stem cells under *in vitro* development Viability of the cultured cells was determined by trypan blue exclusion dye. The data obtained from 7 cord bloods, columns are mean values and bars represent SD. * *p *< 0.05

The viability of the cultured cells on day 8 was 100 % in both conditions as shown in Figure 2(Fig. 2). Viability of the cells started to decline after day 8. TNC reduced by 19 % and 12 % in on day 12 in A and B condition, respectively, as shown trypan blue exclusion. The viability of cells present on days 16, 20, 24 and 28 under B condition were significantly higher than that under A condition (*p* = 0.02). By day 40, most cells were dead; the number of viable cells remaining was 3.2 x 10^5^± 2 x 10^4^and 6 x 10^5 ^± 1.5 x 10^4^ cells in A and B condition, respectively. 

### Phenotypic profiles of the immune cells among total mononuclear cells derived from HSC

**Figure 3:**
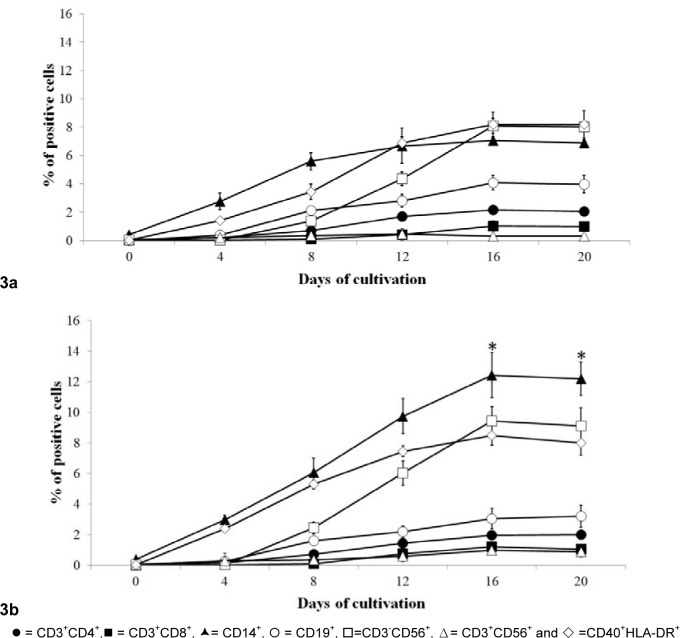
Evolution of phenotypic profiles of the immune cells among total mononuclear cells derived from HSC. Various immune cell types were observed in 2 different culture conditions (3a = condition A and 3b= condition B) in each time point during cultivation period by using flow cytometry. Data obtained from 7 cord bloods are mean with S.D., * =*p*< 0.05

Flow cytometry and various monoclonal antibodies were used to characterize the phenotype upon differentiation of mononuclear cells obtained in A and B conditions. At the first culture time point assessed (day 4, Figure 3(Fig. 3)). MNC *in vitro* differentiation into CD14^+^ cells and CD40^+^HLA-DR^+^ cells was observed. On day 8, the highest phenotype was CD19^+^ cells in both culture conditions. The CD19^+ ^cells population appeared as highest number through day 20 of A condition, whereas in B condition the CD14^+^ cells population was the highest at days 16 and 20.

Comparison between the levels of immune cells obtained from these culture conditions and those in the peripheral blood (PB) are shown in Table 2(Tab. 2). Monocytes and NKcells from the *in vitro* differentiation were similar to those levels *in vivo* as reported from the PB; T lymphocytes and DC were lower than those levels in PB (2.5-fold and 2.9-fold, respectively), whereas B cells and NKT cells were higher than those in PB (3.4-fold and 3.7-fold, respectively). In B condition, number of CD14^+^ cells was higher than that in A condition and the cultivated cells of A and B condition gave a higher number of CD14^+^ cells than that in PB.

### Responsiveness of HSC-derived MNCs and PBMC in response to mitogen

**Figure 4:**
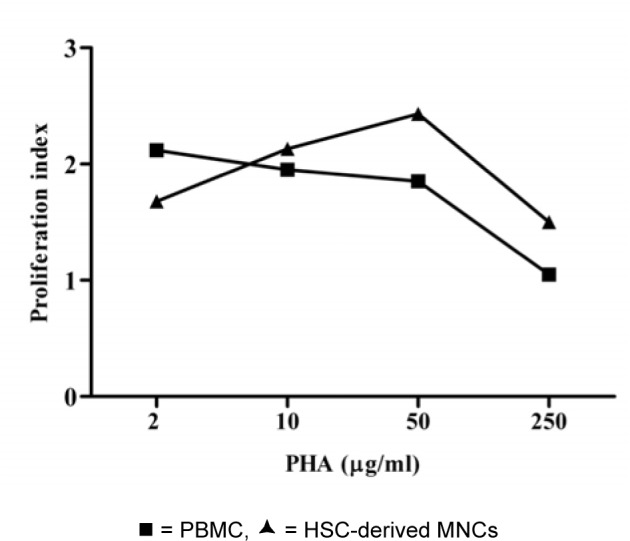
Proliferation indexes of the PBMC and HSC-derived mononuclear cells upon stimulation with PHA. The total numbers of viable nucleated cells in the cultures were measured by trypan blue exclusion on day 4 of the cultivation. Data obtained from 7 cord bloods

To evaluate mitogenic response, PHA was used to stimulate HSC-derived MNCs after 12 days of culture. The MNCs responded well to PHA as shown in Figure 4(Fig. 4). The level of proliferation index upon stimulation of PBMC and HSC-derived MNCs with PHA were similar.

However, this response of HSC-derived MNCs was based on stimulation with 50 µg/ ml PHA while only 2 µg/ml PHA was used for PBMC.

## Discussion

In the present study we enriched and highly purified CD34^+^ cells from umbilical cord blood and characterized various cell types of the immune system formed during the *in vitro* cultivation. The major goal of our study was to determine the patterns of cell proliferation and differentiation in the presence or absence of GM-CSF and G-CSF. These two cytokines are central to hematopoiesis, and the modulation of functional responses, as well as the maintenance of homeostasis and overall immune competence (Möhle and Kanz, 2007[19]). 

SCF, IL-3, transferin and humulin were used to enhance the proliferation, differentiation, and survival of hematopoietic cells. These cytokines are critical for an adequate expansion and development of the hematologic lineages (Duarte and Frank, 2000[10]). Our study demonstrated that the total nucleated cells in the presence of GM-CSF and G-CSF were elevated and exhibited enhanced survival compared with cells in condition A. In addition, GM-CSF and G-CSF prolonged survival and expanded lifespan of cells in blastic stage in the culture, only during the first week of cultivation after which they reverted back to homeostasis. This finding might be the effect of GM-CSF in regulating the expansion and maturation of primitive hematopoietic progenitors (Barreda et al., 2004[2]; Lin et al., 1989[18]). Ultimately, these CD34^+^ cells serve as the only source of progenitor cells for the *in vitro* development of hematopoietic cells.

In this study, we used defined cytokine combinations as the minimal culture supplements to prevent the development of any particular cell lineage of HSC, allowing us to observe the unbiased differentiation of HSC *in vitro*. Various immune cell types including T cells, B cells, monocytes, NK cells, NKT cells and DC cells, were obtained under these conditions. The results indicated that CD34^+^ HSC from cord blood had the ability to produce both lymphoid and myeloid lineages *in vitro*. The *in vitro* production of these two lineages is of great interest in the understanding of immune-biology due to the unique primary responsiveness (considering their naïve status) of these *in vitro* produced mononuclear cells. However, 40-50 % of the remaining cells found in both culture conditions were polymorph nuclear (PMN), making them the major population of WBC as determined by Giemsa staining (data not shown). 

The number of monocytes and NK cells formed in the *in vitro* cultures were similar to the levels found in peripheral blood (Table 2(Tab. 2)). This indicates that the regulation of proliferation and differentiation of HSC in these culture conditions was functioning to control cell growth. This is supported by the notion that the combination of external signals from the bone marrow microenvironment (Can, 2008[5]) and internal factors like transcription factor could strongly influence the development pattern of the hematopoietic stem cells. B cells and NKT cells had greater numbers of cells than PB, showing that these culture conditions support the differentiation of these cells. In addition T cells (CD3^+^ cells) and dendritic cells had lower number of cells than PB, probably because T cells and dendritic cells need other factors such as IL-2 and IL-4, respectively. These cytokines are required for growth and survival and are capable of inducing cell proliferation (Bachmann and Oxenius, 2007[1]).

HSC-derived T lymphocytes in these culture conditions responded to PHA stimulation. This suggested that the derived T lymphocytes had the ability to be directly responsive to mitogens. T lymphocytes derived from HSC required a longer exposure time and higher concentration of PHA to induced mitogenic stimulation than that needed for peripheral blood T lymphocytes. The need for longer exposure at higher concentrations for the *in vitro* produced lymphocytes may be associated with the immaturity of the T cells, since PHA is a potent stimulator of mature T cells (Webb et al., 1973[25]; Stone et al., 2009[22]). In addition, a high lectin binding sites on the CD34^+^ stem cells were recently identified by flow cytometry and light microscopy (Kuemmel et al., 1997[16]). PHA may have absorbed to CD34^+^ cells leading to the need for a greater PHA concentration to activate T lymphocytes.

In summary, GM-CSF and G-CSF have the ability to prolong the survival of the expanding population of HSC-derived MNCs and to maintain the survival of cells in blastic stage. This is the first report showing the complete differentiation and proliferation of immune cells derived from CD34^+ ^HSC under *in vitro* culture conditions. HSC-derived MNCs were capable of responding tomitogen. Lymphocytes, monocytes, dendritic cells and polymorph nuclear cells derived from HSC *in vitro* are unique and thus may benefit various studies such as innate immunity and models of immune disorders.

## Acknowledgements

This project is supported by the Office of the Higher Education Commission, Thailand for funding under the program Strategic Scholarships for Frontier Research Network for the Ph.D. Program, Thai Doctoral degree and Mahidol University under the National Research Universities Initiative; Faculty of Science, Mahidol University and Institutional Research Grant from the Thailand Research Fund (IRG5780011). We wish to thank Research Center, Faculty of Medicine, Ramathibodi Hospital, Mahidol University for technical assistance in flow cytometry; the staff at Department of Obstetrics and Gynaecology, Faculty of Medicine, Ramathibodi Hospital, Mahidol University for the handling of cord blood samples; to Dr. Arther E. Brown and Dr. Sebastain C.P. Bhakdi for English proofing and useful discussion.

## Conflict of interest

There is no conflict of interest.

## Figures and Tables

**Table 1:**
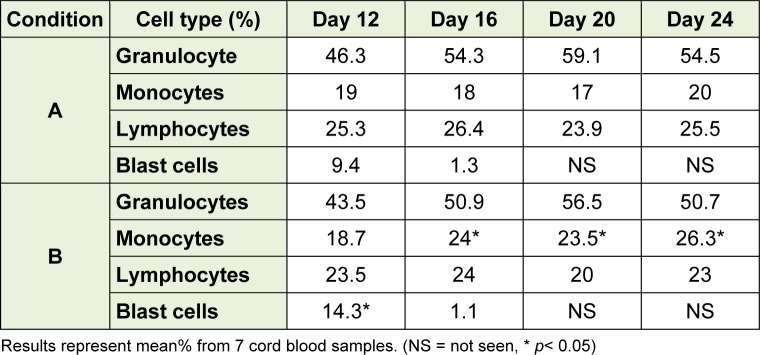
Morphological examination of HSC cultivation in two different culture conditions

**Table 2:**
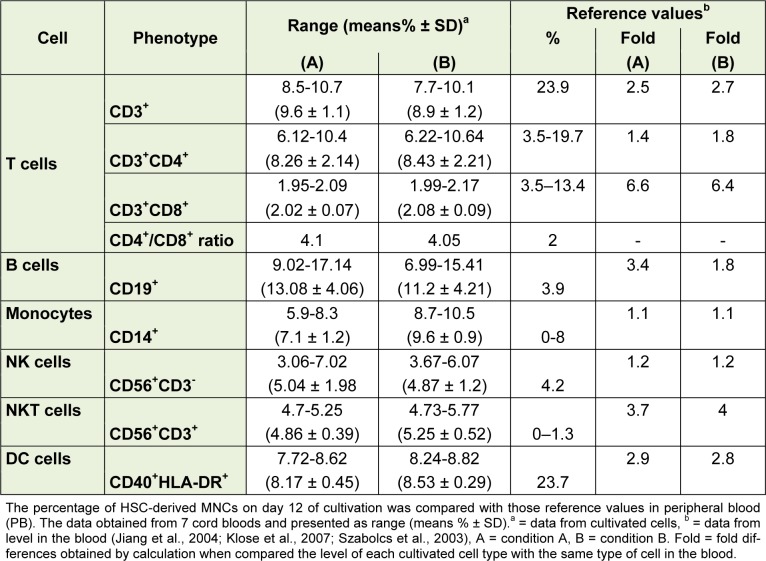
Comparison between HSC-derived mononuclear cells (day 12) under *in vitro* culture conditions with those in peripheral blood
